# Retraction: Resveratrol improves mitochondrial biogenesis function and activates PGC-1α pathway in a preclinical model of early brain injury following subarachnoid hemorrhage

**DOI:** 10.3389/fmolb.2025.1751510

**Published:** 2025-11-28

**Authors:** Frontiers Editorial Office

The journal retracts the 22 April 2021 article cited above.

Following publication, concerns were raised regarding the integrity of the images in the published figures. The authors failed to provide a satisfactory explanation during the investigation, which was conducted in accordance with Frontiers’ policies.

This retraction was approved by the Chief Editors of Frontiers in Molecular Biosciences and the Chief Executive Editor of Frontiers. The authors received a communication regarding the retraction and had a chance to respond. This communication has been recorded by the publisher.

